# A global case meta-analysis of three-dimensional speckle tracking for evaluating the cardiotoxicity of anthracycline chemotherapy in breast cancer

**DOI:** 10.3389/fcvm.2022.942620

**Published:** 2022-09-23

**Authors:** Li Zhang, Rui Zhang, Ping Shuai, Jie Chen, Lixue Yin

**Affiliations:** ^1^Clinical Medicine Academy, Southwest Medical University, Luzhou, China; ^2^Public Health College, Southwest Medical University, Luzhou, China; ^3^Health Management Center of Sichuan Provincial People’s Hospital, Chengdu, China; ^4^Department of Breast Surgery, Sichuan Provincial People’s Hospital, Chengdu, China; ^5^Key Laboratory of Ultrasound in Cardiac Electrophysiology and Biomechanics of Sichuan Province, Institute of Ultrasound in Medicine, Sichuan Academy of Medical Science and Sichuan Provincial People’s Hospital, Chengdu, China

**Keywords:** speckle tracking imaging, breast cancer, anthracycline chemotherapy, cardiotoxicity, meta-analysis

## Abstract

**Background:**

Anthracycline cardiotoxicity has become one of the most common complications of anthracycline therapy. Regular follow-up of chemotherapy patients with myocardial deformation parameters might be helpful for early diagnosis of myocardial damage and protective intervention. This study aimed to investigate the value of three-dimensional speckle tracking imaging (3D-STI) in diagnosing and predicting potential cardiotoxicity in breast cancer patients undergoing anthracycline therapy through meta-analysis based on global cases collection.

**Methods:**

Relevant case-control studies published prior to November 2021 were extracted to assess cardiotoxicity by 3D-STI in breast cancer patients undergoing chemotherapy. Weighted mean difference (WMD) and 95% confidence interval (CI) were used as pooled statistics. Meta regression and subgroup analysis were employed to identify sources of heterogeneity and publication bias was evaluated by Egger’s test and funnel plot.

**Results:**

A total of 1,515 breast cancer patients from 14 studies were enrolled and followed up for 4 or 6 cycles of chemotherapy. Following chemotherapy, absolute values of Left ventricular ejection fraction (LVEF) WMD = –1.59, 95% CI (–1.99, –1.20); *p* < 0.001; global longitudinal strain (GLS) WMD = 2.19, 95% CI (1.87, 2.51); *p* < 0.001; global circumferential strain (GCS) WMD = 1.69, 95% CI (1.11, 2.26); *p* < 0.001; global radial strain (GRS) WMD = –1.72,95% CI (–2.44, –1.00); *p* < 0.001, and global area strain (GAS) WMD = 6.25, 95% CI (4.48, 8.02); *p* < 0.001 were decreased. A medium degree of heterogeneity was shown for values of LVEF (*I*^2^ = 44.4%, *p* = 0.037) while values for GLS (*I*^2^ = 59.0%, *p* = 0.003), GCS (*I*^2^ = 81.3%, *p* < 0.001) and GRS (*I*^2^ = 57.5%, *P* = 0.004) showed a large degree of heterogeneity. Egger’s test and funnel plot showed no significant publication bias in GLS, GCS and GAS data (all *p* > 0.05).

**Conclusion:**

3D-STI has utility for the non-invasive and objective evaluation of changes in left ventricular function in breast cancer patients undergoing chemotherapy with anthracyclines. The current findings have clinical potential for the early evaluation of myocardial injury caused by chemotherapy toxicity.

## Introduction

Anthracyclines constitute a basic chemotherapeutic medicine for breast cancer but cardiotoxicity is the most common and serious adverse effect (1). The resulting myocardial dysfunction often leads to discontinuation of treatment with consequent increased risk of cancer recurrence and mortality. The mortality rate of patients with cancer therapy-related cardiac dysfunction (CTRCD) has been reported to be as high as 40% over the 5 years following chemotherapy (2).

During or within 1 year post-treatment, 2–5% of patients develop early onset chronic progressive cardiotoxicity. This common form of cardiotoxicity usually manifests as dilated cardiomyopathy finally. A further 2–10% of patients will develop late-onset chronic cardiotoxicity 1 year after the completion of treatment and since compensatory mechanisms and physiological reserves maintain normal cardiac function until a threshold point is reached, patients may have no obvious symptoms for a long time after chemotherapy (3). Therefore, early detection of the subclinical cardiotoxicity is critical to the reduction of chemotherapy-related adverse cardiac events (4).

Left ventricular ejection fraction (LVEF) values are widely used in the clinic to assess patients with myocardial toxicity but the accuracy and sensitivity of such values in determining myocardial injury at early stage has been questioned. By the time decreases in LVEF values are severe enough to be observed, myocardial dysfunction is already in a state of advanced deterioration (5). Therefore, there is a pressing need for screening technology to diagnose subclinical dysfunction.

Speckle tracking echocardiography (STE) has been used over the last decade in the non-invasive evaluation of cardiac mechanics and function. Its utility for diagnosis and longitudinal strain measurement following anthracycline treatment has been established (3, 4). Whereas a greater than 15% reduction in post-therapeutic left ventricular longitudinal strain combined with LVEF greater than 50% or less than 50% without reduction more than 10% has been regarded as subclinical myocardial injuries. But, this threshold still may not be sensitive enough to allow early detection of CTRCD (3). Further inclusion of other strain parameters may potentiate the early assessment of myocardial damage in breast cancer patients undergoing chemotherapy.

Three-dimensional speckle tracking imaging (3D-STI) represents an advanced form of echocardiographic technology with utility for the detection of myocardial injuries at earlier stages. 3D-STI allows tracking of out-of-plane speckle motion and has higher measurement reproducibility. Since multi-section images during the same cardiac cycle can be resolved, the evaluation time is reduced.

The current study is a meta-analysis of observational studies of conventional echocardiography and 3D-STI with respect to altered myocardial function during breast cancer chemotherapy. The value of 3D-STI parameters in monitoring of early stage myocardial injuries in the longitudinal, radial and circumferential planes of the left ventricle has been explored.

## Methods

### Data sources and search strategy

English-language databases, Pubmed, Cochrane, Embase and Web of Science, and Chinese databases, CNKI, Wanfang, and VIP were searched for relevant articles published before November 1, 2021. The search terms used were as follows: “echocardiography,” “three-dimensional speckle tracking,” “three dimensional echocardiography,” “three-dimensional ultrasound,” “3-D echocardiography,” “cardiotoxicity,” “speckle tracking,” “anthracycline,” “breast cancer.” Relevant references of the literature were manually searched.

### Three-dimensional speckle tracking imaging

**FIGURE 1:**
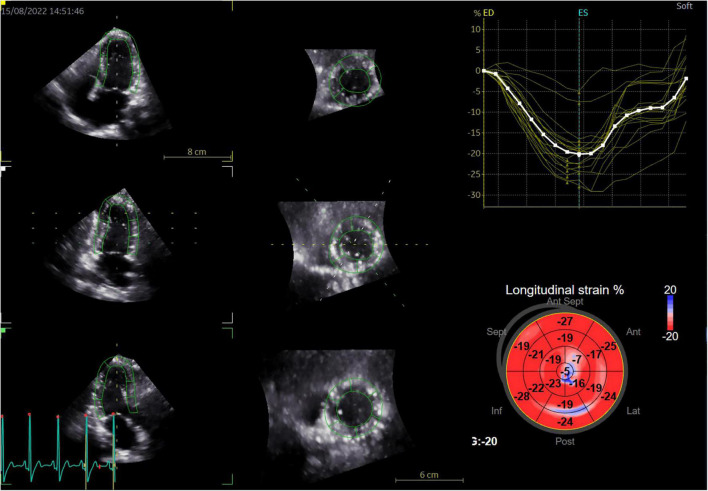
Representative diagram of three-dimensional speckle-tracking strain measurements in a patient with breast cancer treated with chemotherapy (left panel: sections generated by 3D full-volume image cut of the LV, right panel: the color-coded bull’s eye plot of the longitudinal strain in the LV 17 segment and corresponding longitudinal strain regional values).

Three dimensional strain analysis is mainly used to measure myocardial deformation in three-dimensional space. After importing the 3D gray-scale images into the workstation, the system will automatically track the endocardial surface, and the region of interest can be manually adjusted if necessary. The system automatically generates a color-coded 17 segmental illustration of the bull eye and strain parameter curve of each segment (Figure 1). The weighted mean of the regional values from the 17 myocardial segments was also calculated to obtain parameters, including GLS, GCS, GRS, and GAS.

### Inclusion and exclusion criteria

Inclusion criteria were as follows: (1) prospective and retrospective studies using conventional echocardiography and 3D-STI to assess cardiotoxicity in breast cancer patients during chemotherapy; (2) during the completion of anthracycline chemotherapy, adjuvant hormonal therapy, radiotherapy, or other treatments were not performed at the same time; (3) Ultrasound assessment of myocardial toxicity before chemotherapy and after the fourth or sixth cycle of chemotherapy; (4) inclusion of relevant parameters, such as left ventricular ejection fraction (LVEF), global longitudinal strain (GLS), global circumferential strain (GCS), global radial strain (GRS), and global area strain (GAS).

Exclusion criteria were as follows: (1) publications with incomplete data; (2) reviews, conference papers, letters from readers or editorial comments; (3) duplicate publications; (4) publications with unclear chemotherapy regimens; (5) publications with unclear follow-up dates.

### Literature screening and data extraction

Eligible articles were screened according to the inclusion and exclusion criteria by two researchers and the quality scored according to the Newcastle-Ottawa-Scale (NOS) on a scale of 0–9. Literature was selected according to its score and information extracted as follows: first author, study country, publication time, study sample size, chemotherapy protocol, accompanying diseases, echo timing, vendor, workstation, LVEF, GLS, GCS, GRS, and GAS data.

### Statistical analysis

StataSE 15.0 was used for statistical analysis and continuous variables were analyzed according to weighted mean difference (WMD) and 95% confidence interval (CI). *I*^2^ and *Q*-tests were used to assess heterogeneity: high heterogeneity: *I*^2^ > 50% and *p* < 0.05; medium heterogeneity: 25% ≤ *I*^2^ ≤ 50%; low heterogeneity: *I*^2^ < 25%; no heterogeneity: *p* ≥ 0.05 (6). Heterogeneous results were subjected to meta regression and subgroup analysis to explore the source of heterogeneity. Egger’s test and funnel plot were used to evaluate publication bias. Sensitivity analysis was performed by removing sources of significant change from results of a single publication to judge the stability of the results. Statistical significance was shown by *p* < 0.05.

## Results

### Search results and quality evaluation

**FIGURE 2:**
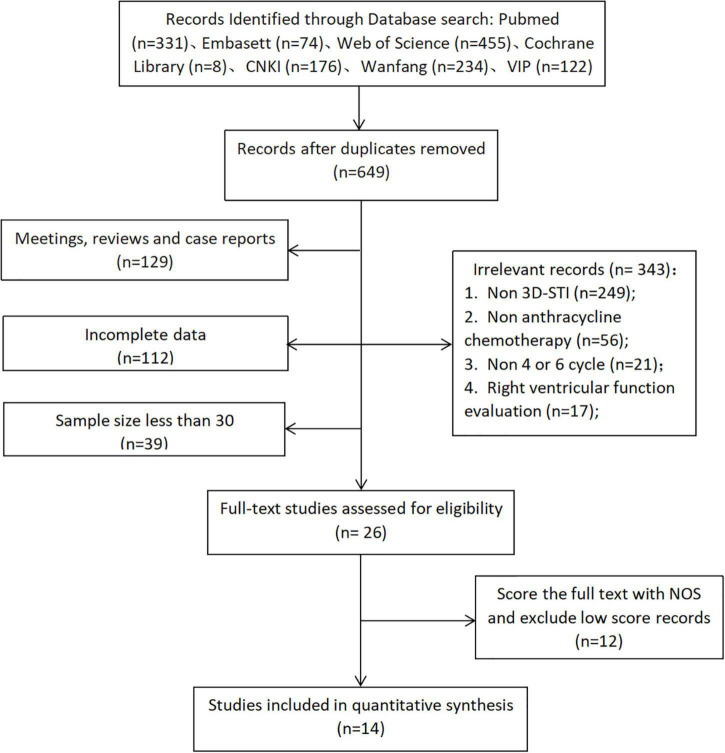
Flow diagram to show study selection (Through literature retrieval in multiple databases, according to the inclusion and exclusion criteria of this study, 14 literatures were finally included into meta-analysis).

A total of 1,399 publications were initially identified of which 14 (7–20), describing 1,515 cases, satisfied the inclusion and exclusion criteria and entered the meta-analysis (Figure 2). These included 6 English and 8 Chinese publications which measured LVEF, GLS, GCS, and GRS parameters and 10 measured GAS parameters. Scoring by the NOS scale indicated 8 high-quality (score ≥ 7) and 6 reliable articles (score ≥ 5; Table 1).

**TABLE 1:** Summary of General information and quality assessment of the included literature.

References	Country	*n*	Age (years)	3D-STI parameter	Chemotherapy protocol	Accompanying diseases	Echo timing	Vendor	Workstation	NOS score
										
Chen et al. (7)	China	83	49.25 ± 8.75	GLS, GCS, GRS, GAS, LVEF	Epirubicin, cyclophosphamide	–	Pre- and 6-cycle post-anthracycline	GE Vivid E9	EchoPAC 110	8
Santoro et al. (8)	Italy	100	48.60 ± 11.10	GLS, GCS, GRS, GAS, LVEF	Fluoruracile, epirubicin, cyclophosphamide	–	Pre- and 6-cycle post-anthracycline	GE Vivid E9	EchoPAC 110.1.1	6
Piveta et al. (9)	Israel	47	48.70 ± 10.80	GLS, GCS, GRS, GAS, LVEF	Anthracyclines	–	Pre- and 4-cycle post-anthracycline	GE Vivid E9	–	7
Ruan et al. (10)	China	60	53.20 ± 9.60	GLS, GCS, GRS, GAS, LVEF	Epirubicin, cyclophosphamide	–	Pre- and 4-cycle post-anthracycline	GE Vivid E9	EchoPAC 201	8
Wang et al. (11)	China	46	—	GLS, GCS, GRS, GAS, LVEF	Anthracyclines	–	Pre- and 4-cycle post-anthracycline	GE Vivid E95	EchoPAC	6
Chen et al. (12)	China	63	47.60 ± 8.26	GLS, GCS, GRS, GAS, LVEF	Doxorubicin, cyclophosphamide	–	Pre- and 4-cycle post-anthracycline	GE Vivid E9	EchoPAC 110	7
Chen et al. (13)	China	83	49.25 ± 8.18	GLS, GCS, GRS, GAS, LVEF	Epirubicin, cyclophosphamide	–	Pre- and 6-cycle post-anthracycline	GE Vivid E9	Echopac 110.1.1	7
McGowan et al. (21)	China	50	51.60 ± 9.60	GLS, GCS, GRS, LVEF	Epirubicin, cyclophosphamide	–	Pre- and 6-cycle post-anthracycline	GE Vivid E9	EchoPAC	8
Stoodley et al. (15)	Australia	52	49.00 ± 9.00	GLS, GCS, GRS, LVEF	Doxorubicin (77%), epirubicin (23%)	Hypercholesterolemia 21%, hypertension 25%, diabetes 4%, Ischemic heart disease 6%	Pre- and 6-cycle post-anthracycline	GE Vivid 7	EchoPAC 6.0	6
Xue et al. (16)	China	46	50.80 ± 10.40	GLS, GCS, GRS, LVEF	Anthracyclines	–	Pre- and 6-cycle post-anthracycline	Philips IE33	Qlab8.0	7
Chen et al. (17)	China	60	57.60 ± 8.26	GLS, GCS, GRS, GAS, LVEF	Epirubicin, cyclophosphamide	–	Pre- and 4-cycle post-anthracycline	GE Vivid95	EchoPAC 201	6
Zhang et al. (18)	China	32	49.00 ± 6.00	GLS, GCS, GRS, GAS, LVEF	Fluoruracile, epirubicin, cyclophosphamide	–	Pre- and 4-cycle post-anthracycline	Toshiba Artida SSH-880 CV	–	7
Zhang et al. (19)	China	40	44.00 ± 10.00	GLS, GCS, GRS, GAS, LVEF	Doxorubicin, cyclophosphamide	–	Pre- and 4-cycle post-anthracycline	GE Vivid E9	EchoPAC	6
Sawaya et al. (20)	USA	43	49.00 ± 10.00	GLS, GCS, GRS, LVEF	Doxorubicin (91%), epirubicin (9%)	Hypertension 28%, hypercholesterolemia 19%, diabetes 2%	Pre- and 4-cycle post-anthracycline	GE Vivid 7/E9	EchoPAC	6

“-,” Means not mentioned in the literature; Data are presented as mean ± standard deviation; GLS, global longitudinal strain; GCS, global circumferential strain; GRS, global radial strain; GAS, global area strain; LVEF, left ventricular ejection fraction.

### Literature analysis

**FIGURE 3:**
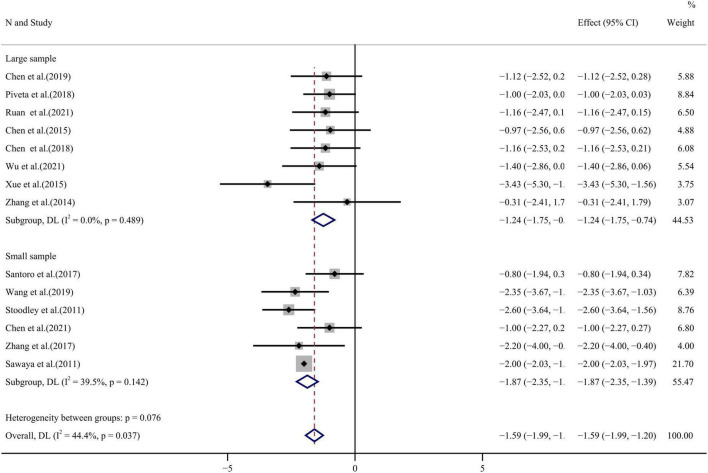
LVEF forest plot [Forest plot of weighted mean difference (WMD) of the association between LVEF absolute value reduction and anthracyclines therapy].

**FIGURE 4:**
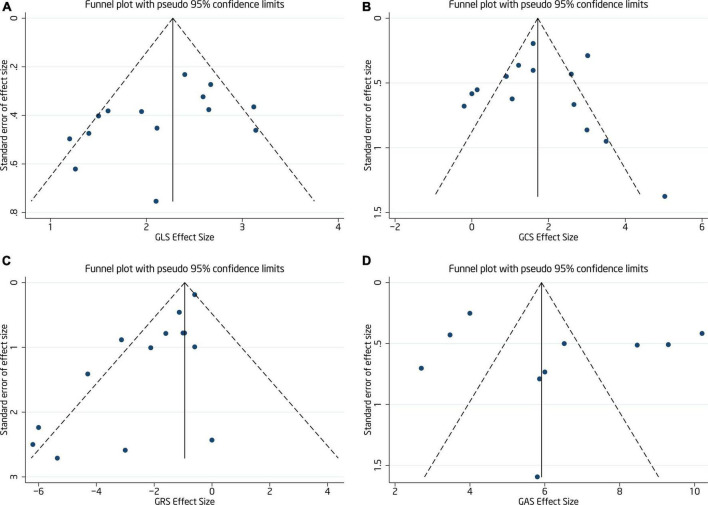
Funnel plot of GLS **(A)**, GCS **(B)**, GRS **(C)**, and GAS **(D)** (The symmetry of funnel plot was used to evaluate the publication bias of included studies).

Fourteen publications compared LVEF values before and after chemotherapy with moderate heterogeneity among the studies (*I*^2^ = 44.4%, *p* = 0.037). Analysis by random effect model found decreased LVEF after anthracycline chemotherapy WMD = –1.59, 95%CI (–1.99, –1.20), *p* < 0.001 (see forest plot in Figure 3). Egger’s test and funnel plot distribution (Figure 4) showed a degree of publication bias (*p* = 0.045).

**FIGURE 5:**
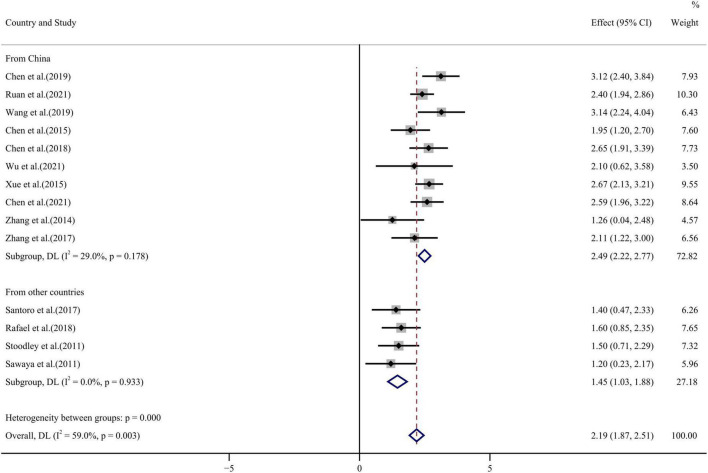
GLS forest plot [Forest plot of weighted mean difference (WMD) of the association between GLS absolute value reduction and anthracyclines therapy].

GLS values before and after chemotherapy showed a high degree of heterogeneity among 14 publications (*I*^2^ = 59.0%, *p* = 0.003) and random effect model analysis found lower values following chemotherapy than before WMD = 2.19, 95% CI (1.87, 2.51), *p* < 0.001 (see forest plot in Figure 5). Egger’s test and funnel plot (Figure 4) showed symmetrical distribution of GLS values with no publication bias (*p* = 0.108).

**FIGURE 6:**
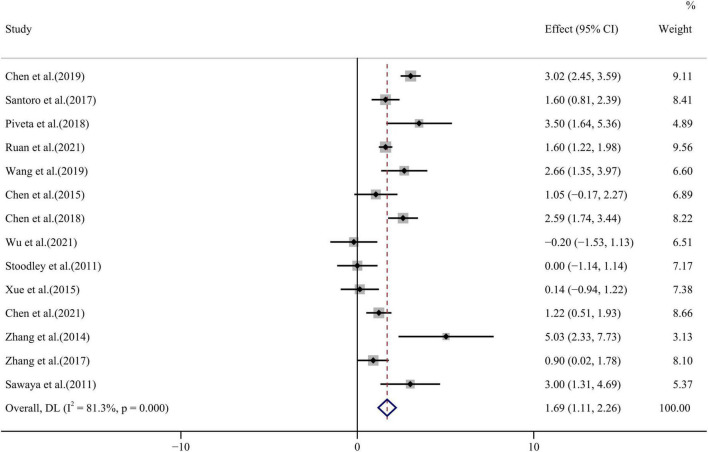
GCS forest plot [Forest plot of weighted mean difference (WMD) of the association between GCS absolute value reduction and anthracyclines therapy].

All 14 papers evaluated differences in GCS values before and after anthracycline chemotherapy and with highly heterogeneous results (*I*^2^ = 81.3%, *p* < 0.001). The forest plot of GCS WMD = 1.69, 95% CI (1.11, 2.26), *p* < 0.001 is shown in Figure 6. Egger’s test and funnel plot distribution (Figure 4) indicated no publication bias (*p* = 0.978).

**FIGURE 7:**
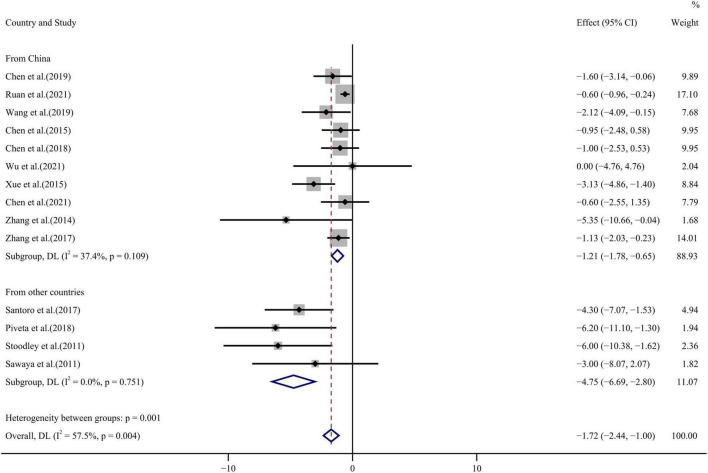
GRS forest plot [Forest plot of weighted mean difference (WMD) of the association between GRS absolute value reduction and anthracyclines therapy].

Similarly, all 14 publications reported GRS values before and after chemotherapy with some heterogeneity among the results (*I*^2^ = 57.5%, *p* = 0.004) which were analyzed by a random effect model. GRS WMD = –1.72, 95%CI (–2.44, –1.00), *p* < 0.001 decreased significantly after chemotherapy (see Figure 7 for forest plot). Some degree of publication bias was evident from Egger’s test and funnel plot distribution (*p* = 0.001) (Figure 4).

**FIGURE 8:**
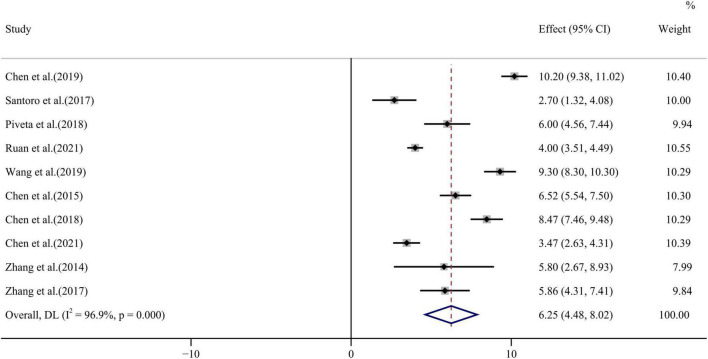
GAS forest plot [Forest plot of weighted mean difference (WMD) of the association between GAS absolute value reduction and anthracyclines therapy].

GAS values before and after chemotherapy were compared by 10 publications with high heterogeneity among the studies (*I*^2^ = 96.9%, *p* < 0.001). Analysis by random effect model showed that GAS values WMD = 6.25, 95% CI (4.48, 8.02), *p* < 0.001 decreased after chemotherapy (see Figure 8 for forest plot). No publication bias was seen by Egger’s test and funnel plot distribution (*p* = 0.499) (Figure 4).

### Meta regression and subgroup analysis

Sources of heterogeneity were explored by meta regression analysis. Regression model covariates were set as follows: (1) Sample size: ≥ 60: 1, < 60: 0; (2) Study country: China: 1, elsewhere: 0; (3) Chemotherapy cycle: 4 cycles: 1, 6 cycles: 0; (4) instrument and equipment type: GE vivid series: 1, non GE vivid series: 0; (5) Quality of included literature (NOS scores): ≥ 7: 1, ≥ 5: 0.

Meta regression analysis showed that LVEF heterogeneity arose from sample size, that of GLS and GRS from the source country and that of GAS from sample size, chemotherapy cycle and study country (all *p* < 0.05). The relationship between the source of GCS heterogeneity and the above covariates did not achieve statistical significance (Table 2).

**TABLE 2:** Strain parameter of meta regression analysis results.

Variable	*P*-value (LVEF)	*P*-value (GLS)	*P*-value (GCS)	*P*-value (GRS)	*P*-value (GAS)
*n*	0.039	0.696	0.585	0.417	0.035
Cycle	0.301	0.136	0.188	0.400	0.049
Quality	0.109	0.896	0.444	0.935	0.131
Equipment	0.859	0.399	0.670	0.249	0.182
Country	0.368	0.007	0.374	0.014	0.046

Publications were classified according to the factors affecting heterogeneity in the meta regression results and subgroup analysis was conducted. Subgroup analysis showed that LVEF heterogeneity arose from sample size and inclusion of only the large sample group reduced heterogeneity to zero. GLS and GRS heterogeneity arose from the study country of origin and was improved by inclusion of literature from countries other than China. No source of heterogeneity could be identified from analysis of the GAS subgroup.

### Sensitivity analysis

Sensitivity analysis was conducted by sequential elimination of individual articles. Values for LVEF, GLS, GCS, GRS, and GAS did not change significantly, indicating a good degree of stability.

## Discussion

Advances in anti-tumor chemotherapy research have led to huge improvements in breast cancer survival rates. However, CTRCD remains the most common complication of anthracycline therapy, attracting the attention of clinicians, and affecting overall patient prognosis.

It is thought that anthracycline cardiotoxicity arises with the inhibition of topoisomerase 2β which activates the cell death pathway and reduces mitochondrial biogenesis. Cumulative dosage of anthracyclines, age, preexisting fundamental cardiovascular disease, atrial fibrillation, hypertension, diabetes, and smoking history are all risk markers for cardiotoxicity (21).

Patients’ left ventricular function is often monitored clinically by means of LVEF. A decrease in LVEF of > 10% or a value of LVEF of < 50% post-chemotherapy is considered to indicate cardiotoxicity (22). However, the accuracy and sensitivity of LVEF in determining early myocardial injury is limited. By the time that LVEF values have decreased, cardiac injury has reached a stage which cannot be completely reversed (23, 24). Two-dimensional GLS has previously been introduced as an index of chemotherapy-related cardiotoxicity in the 2016 position paper (3), and many studies have confirmed that the post-chemotherapy reduction in two-dimensional GLS compared with the pre-chemotherapy situation (25–27). However, the role of 2-dimensional longitudinal strain in detecting left ventricular dysfunction remains controversial. Three-dimensional speckle tracking technology overcomes the problems of angle dependence in two-dimensional plane tracking, and is expected to generate more sensitive parameter indicators for the early detection of subclinical myocardial dysfunction.

3D-STI strain parameters reflect myocardial deformation in longitudinal, radial and circumferential spatial directions. GLS represents myocardial deformation from the base to the apex, reflecting contraction of the longitudinal fibers under the endocardium. Longitudinal myocardial movement is central to contraction of the left ventricle. GCS represents the circumferential shortening of left ventricular myocardial fibers in the short axis. GRS reflects deformation toward the center of the left ventricular cavity, indicating thickening and thinning of the left ventricular wall during the cardiac cycle. GAS is the product of longitudinal and circumferential strain, as the left ventricular endocardial surface deforms during systole and diastole. It reflects the change in relative area, combining longitudinal, and circumferential shortening effects (28).

The current meta-analysis found that WMDs of LVEF in breast cancer patients after chemotherapy were lower than those prior to chemotherapy. This suggests a reduction in patients’ ventricular systolic function post-chemotherapy. A previous study (22) has shown a rate of cardiac insufficiency of 9% after anthracycline chemotherapy. Moreover, Zhang et al. (29) found no decrease in breast cancer patients’ LVEF by the second and fourth cycles of chemotherapy but the sixth cycle reduced LVEF compared with values before chemotherapy and compared with those of a control group. Additional studies (30, 31) found no change in routine echocardiographic parameters during breast cancer chemotherapy follow-up, presumably related to drug dosages, and the later onset of reduced LVEF appearance.

The current study found lower absolute values of the 3D-STI parameters, GLS, GCS, GRS and GAS, after chemotherapy, which may predict the development of anthracycline cardiotoxicity. Most publications produced consistent results with only two exceptions. Ruan et al. (10) reported stable GRS values post-chemotherapy while GCS values differed significantly compared with other studies. The findings of the current study reached the opposite conclusion. Wang et al. (11) also reported stable GRS values. It may be that decreases in GLS allow GCS, which reflects circumferential fiber motion, to compensate in order to maintain left ventricular pumping function (10, 32). The precise mechanisms are not known and most studies found decreases in both GLS and GCS while LVEF was maintained.

The decrease in GLS was the most consistent parameter throughout the 14 publications and it has been previously remarked that this parameter may be the earliest to show abnormalities (33). It may be that the large impact of blood flow on the endocardial surface, the abundant subendocardial microcirculation and the relatively high blood drug concentrations make subendocardial fibers more susceptible to ischemia or toxic damage (34). Chang et al. (35) demonstrated the validity of this notion in an animal model of cardiotoxicity. The degree of myocardial fibrosis in the subendocardial layer was shown to be higher than that in the subepicardial layer after doxorubicin treatment by Masson’s trichrome staining. More apoptotic cells were shown to be present in the subendocardium than in the subepicardium by Tunel assay.

There may also be segmental differences in cardiotoxicity. Coutinho Cruz et al. (36) found 24 cases of cardiotoxicity with more obvious damage to the anterior wall, inferior wall and septum of the left ventricle among 105 breast cancer patients. Anqi et al. (37) proposed that the myocardial segment supplied by the left anterior descending coronary artery is more sensitive to damage by anthracyclines. Mechanisms underlying segmental differences are not known but may be related to differential effects of shear stress and left ventricular geometry, plus increased anthracycline exposure in end-stage circulation regions, fibrosis or local variations in apoptosis activation (38).

Some publications cited herein stress the value of GAS in predicting early post-anthracycline myocardial injury due to its inclusion of both longitudinal and circumferential strain and its sensitivity to local cardiac deformation. Zamorano et al. (3) found reduced GAS after chemotherapy and a negative correlation with serum troponin T (Hs-cTnT). Receiver operating characteristic curves for GAS parameters reported by Stoodley et al. (15) give an area under the curve of 0.897. For a –30.55% cut-off point, sensitivity was 0.857 and specificity, 0.917. Similar results were also reported by Han et al. (39). Three-dimensional GRS and GCS percentage changes have also been suggested to be good early independent predictors of CTRCD (36). However, the current meta-analysis found large heterogeneities for GCS and GAS which reduces the reliability of statistical conclusions. Such findings may be due to the limited angle of the short axis direction during measurement. The calculation of GAS from a combination of GLS and GCS further affects results. In addition, the reproducibility of GCS, GLS, and the overall GAS value is poor. Many other studies (40, 41) reported insignificant or late-onset post-chemotherapy changes in GRS and GCS making quantification of changes most challenging.

The biggest limitation of 3D-STI is its reliance on image quality which is affected by chest radiotherapy, mastectomy and breast implant surgery, all of which create challenging acoustic windows and reduce image quality. Furthermore, the frame rate of real-time 3D echocardiography is too low to accurately capture all phases of the cardiac cycle. The result is the underestimation of 3D speckle-tracking echocardiography-derived strain measurements (8). Thus, the degree of standardization of 3D-STI is limited.

One limitation of the current study is the inevitable degree of publication bias. Most eligible studies were conducted in China and ethnic and regional variations need to be considered. Moreover, many studies did not carry out long-term follow-up leading to under-observation of chronic delayed cardiac toxicity. Not all 3D STI-related parameters were reported in the studies, so the number of patients included in this meta-analysis was relatively small. Because of the limited evaluation of left ventricular rotation, torsion and strain rate parameters, the relevant parameters were not included in this study. In addition, data allowing analysis of the relationship between anthracycline type or cumulative dose and subsequent reduction of STI parameters was incomplete. Moreover, the combination of anthracyclines with other drugs were not analyzed.

In summary, this meta-analysis presents a further evidence for the early detection of changes in myocardial function in breast cancer patients undergoing anthracycline chemotherapy by the 3D-STI myocardial deformation parameter, which may prove to be a new monitoring method to facilitate prognosis for chemotherapy patients. However, the predictive and diagnostic threshold of this parameter for post-chemotherapy myocardial toxicity still needs long-time follow-up and more multicenter studies.

## Data availability statement

The original contributions presented in this study are included in the article/supplementary material, further inquiries can be directed to the corresponding author.

## Author contributions

LZ and RZ were responsible for the concept and design, jointly consulting and reviewing the literature, and conducting data analysis and data interpretation. PS and JC were responsible for drafting or critically revising the manuscript of the research content and putting forward constructive opinions. LY gave final approval to the submitted manuscript. All authors contributed to the article and approved the submitted version.
